# The Mitotic and Metabolic Effects of Phosphatidic Acid in the Primary Muscle Cells of Turbot (*Scophthalmus maximus*)

**DOI:** 10.3389/fendo.2018.00221

**Published:** 2018-05-04

**Authors:** Tingting Wang, Xuan Wang, Huihui Zhou, Haowen Jiang, Kangsen Mai, Gen He

**Affiliations:** ^1^Key Laboratory of Aquaculture Nutrition, Ministry of Agriculture, Ocean University of China, Qingdao, China; ^2^Key Laboratory of Mariculture, Ministry of Education, Ocean University of China, Qingdao, China; ^3^Laboratory for Marine Fisheries Science and Food Production Processes, Qingdao National Laboratory for Marine Science and Technology, Qingdao, China

**Keywords:** phosphatidic acid, turbot, cell proliferation, target of rapamycin pathway, metabolism

## Abstract

Searching for nutraceuticals and understanding the underlying mechanism that promote fish growth is at high demand for aquaculture industry. In this study, the modulatory effects of soy phosphatidic acids (PA) on cell proliferation, nutrient sensing, and metabolic pathways were systematically examined in primary muscle cells of turbot (*Scophthalmus maximus*). PA was found to stimulate cell proliferation and promote G1/S phase transition through activation of target of rapamycin signaling pathway. The expression of myogenic regulatory factors, including *myoD* and *follistatin*, was upregulated, while that of *myogenin* and *myostatin* was downregulated by PA. Furthermore, PA increased intracellular free amino acid levels and enhanced protein synthesis, lipogenesis, and glycolysis, while suppressed amino acid degradation and lipolysis. PA also was found to increased cellular energy production through stimulated tricarboxylic acid cycle and oxidative phosphorylation. Our results identified PA as a potential nutraceutical that stimulates muscle cell proliferation and anabolism in fish.

## Introduction

Fish grow in unique features among vertebrates. Specifically, most fish grow indeterminately and a significant portion of muscle fibers was formed during juvenile period through long-lasting hyperplastic growth (form more muscle cells) (1, 2) in addition to hypertrophic growth (increase in muscle fiber size), which is the predominant pattern in other vertebrates postnatally (3). This is both of biological interest and economical importance for commercial aquaculture. However, understanding the underlying mechanism of fish muscle cell growth and proliferation is still very limited.

Cell proliferation and growth are tightly regulated by extracellular cues and intracellular metabolites (4). Cells uptake and utilize extracellular nutrients to provide energy and building blocks for proliferation (5), while growth factors provide stimulus to switch between resting and proliferative states (6). Intracellular kinases such as target of rapamycin (TOR) sense the nutritional status and direct how biosynthetic intermediates are used and whether the cell should arrest, grow, or divide (7–10). The molecular integrations of nutrient, biosynthetic intermediates, and energy balance to the core cell cycle machinery have been the focus of many studies (4, 11, 12). TOR, the mitogen-activated protein kinase/extracellular signal-regulated protein kinase (9, 13–15), and phosphoinositide 3-kinase (PI3K)–Akt pathways were found to be involved in cell proliferation and growth control (16–18). The changes of these signaling pathways regulate the levels and activities of cyclins and cyclin-dependent kinases (CDKs), which phosphorylate retinoblastoma protein (Rb) and push the cell into S phase of the division cycle (19–21).

In fish, studies have demonstrated that growth hormone/insulin-like growth factor (IGF) and amino acids stimulated myocyte proliferation in gilthead sea bream, Atlantic salmon, and rainbow trout (22–30). IGF1 receptors express at high levels in muscle tissues and their downstream actions are mainly mediated through PI3K–Akt pathway in fish (17). TOR signaling plays a critical role in sensing intracellular amino acid levels and accretion of myofibrillar proteins in rainbow trout and fine flounders (31, 32). Its inhibition decreased muscle and body growth in fine flounder and turbot (33, 34). However, studies are still very rare in the identification of dietary supplements that can modulate the related pathways and promote fish growth.

Phosphatidic acid (PA) is one of the most important glycerophospholipids found in biomembranes (35). PA has different roles in the cell. For example, it is a precursor for other lipids such as phosphatidylserine or phosphatidylcholine (36). It also influences membrane curvature and acts as signaling lipid (35). PA can be either synthesized endogenously by enzymes such as phospholipase D (37) or obtained from food supplements such as soy lecithins (38). Oral administration of PA stimulated muscle protein synthesis in rat (39) and enhanced muscle thickness in resistance-trained individuals (36). Studies have shown that PA can directly bind and activate TOR and induce muscle fiber hypertrophy in mammals (36, 40, 41). It will be particularly interesting to examine the effects of PA on fish species such as turbot, whose growth is predominantly hyperplastic during juvenile stage (42). Thus, the aim of this research is to systematically examine the effects of PA on cell proliferation and metabolism in turbot primary muscle cells.

## Materials and Methods

### Primary Culture of Turbot Muscle Cells

All animal care and handling procedures in this study were approved by the Animal Care Committee of Ocean University of China. The turbot primary cell culture was generated as described before (43, 44). For each culture, 6–10 turbots weighed approximately 10–15 g were sacrificed and immersed in 75% alcohol for 30 s, and then rinsed by sterilized Dulbecco’s phosphate-buffered saline (DPBS) with 400 U/ml penicillin and 400 µg/ml streptomycin (GIBCO: 15240–112). White dorsal muscle was excised and collected in cold Leibowitz’s L-15 Medium (Sigma: L5520) with antibiotics. The tissue was cut into 1.0 mm^3^ pieces using sterilized stainless steel scissors and forceps. The tissue pieces were centrifuged at 300 *g* for 1 min to collect the pellet and then resuspended in L-15 medium. This operation was repeated for three times and then the pellet was resuspended in 0.1% trypsin in L-15medium. Enzymatic digestion was performed for 10 min at room temperature with gentle agitation and centrifuged for 1 min at 300 *g*. The supernatant was diluted with cold L-15 medium with 10% serum to block trypsin activity. The tissue fragments were subjected to a second trypsin digestion. The supernatants were pooled, filtered through a 100-µm nylon cell strainer, and centrifuged at 300 *g* for 20 min. The pellet was resuspended in growth medium [L-15 medium with 10% serum, 1% antibiotics (penicillin 100 U/ml, streptomycin 100 µg/ml) with 20 mM HEPES, 2 mM GlutaMAX™, and 2.5 ng/ml fibroblast growth factor. The medium osmolarity is 398–420 mOsm/kg]. The cell culture was maintained at 24°C in a humidified incubator without CO_2_. Observations of morphology were regularly made to monitor the status of the cells.

### Cell Treatment

Cells were seeded at a density of 10^6^ cells/ml for experiments except cell proliferation analysis. After 2 days of culture, the cells were incubated in serum-starved medium for 12 h and treated with experimental mediums for 16 h before lysis. PA was bought from Avanti (Cat. no. 840074). A stock solution (10 mM) in DPBS was prepared by sonication as described elsewhere (45, 46). The serum-starved medium was prepared with 20 mM HEPES, 2 mM GlutaMAX™ in L-15 medium and the experimental medium was prepared with 20 mM HEPES, 2 mM GlutaMAX™ in L-15 medium with indicated concentrations of PA. All cell culture experiments were repeated at least three times.

### Cell Proliferation Analysis

Mitogenic response of cells to PA was determined by the CyQUANT™ Cell Proliferation Assay Kit (Molecular Probes, USA) (Cat. no. 7026) according to the manufacturer’s instructions. Cells (2 × 10^4^ cells/well) were seeded in 96-well black plates (Corning Inc.) and treated with various concentrations of PA at 0, 2, 5, 10, and 50 µM for 16 h. The cell medium was then removed and the cell pellet was stored at −80°C. For the assay, cells were lysed, and total cellular nucleic acid was measured using florescence at 520 nm emission after excitation at 480 nm with a fluorometer (spectraMax i3x, Molecular Devices, USA). Each treatment was repeated for three times.

### Cell Cycle and Newly Synthesized DNA Analysis

Cell cycle and newly synthesized DNA were analyzed based on the methods described before (47, 48) with some modifications. Briefly, after treatment, cells were washed twice with DPBS and fixed with 75% cold ethanol at 4°C overnight. Afterward, cells were centrifuged at 200 *g* for 5 min and resuspended with 1 ml DPBS, followed by incubation with 50 µg/ml RNaseA (Invitrogen, USA) (Cat. no. 1692412), and 50 µg/ml propidium iodide (PI) (Life Technologies™, USA) (Cat. no. 3566) for 30 min. For newly synthesized DNA analysis, cells were incubated with 10 µM 5-ethynyl-2′-deoxyuridine (EdU) and processed according to the manufacturer’s instructions of Click-iT™ Plus EdU Alexa Fluor™ Flow Cytometry Assay Kit (Invitrogen, USA) (Cat. no. 10634) and proceed to the next step for staining the cells for DNA content by PI. To examine the newly synthesized DNA, double staining was conducted using PI with EdU, which was incorporated into DNA during active DNA synthesis and coupled to fluorescent dye that can be detected by different excitation and emission filter from PI (49). Cell cycle and newly synthesized DNA was determined by flow cytometry (Beckman Coulter FC 500 MPL).

### Nonradioactive Measurements of Protein Synthesis With SUnSET

Turbot primary muscle cells were cultured and treated as described above. Protein synthesis was analyzed based on the methods described before with some modifications (50). Briefly, during the last 30 min of the treatment, cells were changed to experimental mediums with 1 µM puromycin (Thermo Fisher Scientific, USA, A1113802). The cells were then harvested and analyzed by Western blot with antibody against puromycin (Millipore, USA, MABE343).

### Measurement of Intracellular Free Amino Acids

The concentrations of intracellular free amino acids were measured based on the methods described before (44). Briefly, after PA treatment, cells were rapidly washed with cold DPBS twice and chilled with 2 ml cold methanol on dry ice. Cells were collected with a rubber tipped cell scraper and transferred to liquid nitrogen for 10 min, thawed on ice for 10 min, and briefly vortexed. This freeze–thaw cycle was repeated three times for complete cell disruption. The samples were centrifuged at 4°C at 3,000 *g* for 30 min and the supernatant was collected. The pellet was lysed in 1 ml of lysis buffer (50 mM Tris, 150 NaCl, 0.5% Nonidet P-40, 0.1% SDS, 1 mM EDTA, pH 7.4, with protease inhibitor cocktail) and the protein concentration was quantitated using a BCA Protein Assay Kit (Beyotime Biotechnology) according to the manufacturer’s instructions. After sample normalization based on total protein levels, the collected supernatant was de-proteinized by mixing with equal volume of 10% sulfosalicylic acid and incubated on ice for 15 min. After centrifugation at 15,000 *g* for 15 min, 1 ml of supernatant was filtered through 0.22-µm filters for free amino acid measurement using an automatic amino acid analyzer (L-8900, HITACHI, Japan).

### Western Blot Analysis

Cells were rinsed twice with ice-cold DPBS and lysed in ice-cold RIPA buffer composed of 50 mM Tris, 150 mM NaCl, 0.5% Nonidet P-40, 0.1% SDS, 1 mM EDTA, pH 7.4, with protease and phosphatase inhibitor cocktail (Roche). The cell lysate was collected after centrifugation at 12,000 *g* for 20 min at 4°C. Protein concentration was determined by a BCA Protein Assay Kit according to the manufacturer’s instructions. After normalization, samples (10 µg of protein) were separated by 12% SDS-PAGE gel for 1 h at 150 V and then transferred to a 0.45-µm polyvinylidene difluoride membrane (PVDF) (Millipore) for 1 h at 100 V. After transfer, the membrane was blocked with 5% nonfat milk in TBST for 1 h and incubated with primary antibodies overnight at 4°C, followed by secondary antibodies for 1 h, and developed with ECL Reagents (Beyotime Biotechnology). Antibodies against S6 kinase (Cat. no. 9202), phospho-S6k (Thr389) (Cat. no. 9205), Akt (Cat. no. 9272), phospho-Akt (Ser473, Ser308) (Cat. no. 9271,9275), S6 (Cat. no. 2217), phospho-S6 (Ser240/244), mTOR (Cat. no. 2972), phospho-mTOR (Thr2448), Erk (Cat. no. 9102), phospho-Erk (Cat. no. 9101), 4EBP1 (Cat. no. 9644), phospho-4EBP1 (Thr37/46) (Cat. no. 9459), phosphor-eIF2α (Cat. no. 3597), eIF2α (Cat. no. 9722), Rb (Cat. no. 9313), phospho-Rb (Ser807/811) (Cat. no. 8516), and β-tubulin (Cat. no. 2148) were from Cell Signaling Technology Inc. Cdk2 (Cat. no. 6248), Cyclin E (Cat. no. 48420), and minichromosome maintenance protein 2 (Mcm2) (Cat. no. 373702) antibodies were from Santa Cruz Biotechnology Inc. All these antibodies were developed using antigenic regions completely conserved in turbot, and many had been successfully used in turbot as reported before (34, 44, 51). All experiments were repeated at least three times. The densities of the protein bands were normalized to that of β-tubulin, which served as an inner control. All the band intensities were quantified using NIH Image 1.63 software.

### Quantitative Real-Time PCR (qRT-PCR)

For qRT-PCR, cells were lysed with Trizol Reagent (Invitrogen, USA) according to the manufacturer’s instructions. The extracted RNA was quantified by Nanodrop 2000 spectrophotometer (Thermo Fisher Scientific, USA), and the integrity was examined by 1% denatured agarose gel electrophoresis. The genomic DNA was removed and single-stranded cDNA was synthesized using a PrimeScript^®^ RT Reagent Kit with gDNA Eraser (Takara, Japan). The qRT-PCR analysis was conducted as described (52). All primer sequences of target genes were listed in Table S1 in Supplementary Material and many had been published previously (34, 44, 51, 53). To calculate the expression levels of target genes, results were normalized to *RNA polymerase II subunit D* (*rpsd*), as no expression changes of *rpsd* were observed in turbot primary muscle cells among treatments. The qRT-PCR was carried out in a quantitative thermal cycler Mastercycler ep realplex (Eppendorf, Germany). SYBR Green Real-Time PCR Kit (Takara, Japan) was used. The program was set at 95°C for 5 min, followed by 40 cycles of 95°C for 5 s, 58°C for 15 s, and 72°C for 20 s, and a final extension step at 72°C for 5 min. The melting curve was performed after the amplification phase for confirmation of the product specificity. Target gene expression levels were quantitated by the 2^−ΔΔCt^ method (54). The data were reported as fold increase of the control (without PA stimulation).

### Statistical Analysis

Each value is expressed as mean ± SEM. All statistical evaluations were analyzed using the software SPSS 22.0.0.0. Comparison of data from two treatments was performed by independent samples *t* tests. The effects of PA and rapamycin treatment and their interactions were analyzed by two-way ANOVA. Tukey’s multiple range tests were used to examine treatment differences among the interactions. When the interaction was significant, the results were further analyzed using one-way ANOVA and Turkey’s multiple range test. Prior to the statistical tests, data were examined for homogeneity of variances (*F*-test). Differences were regarded as significance when *P* < 0.05.

## Results

### PA Induced Cell Proliferation

**Figure 1:**
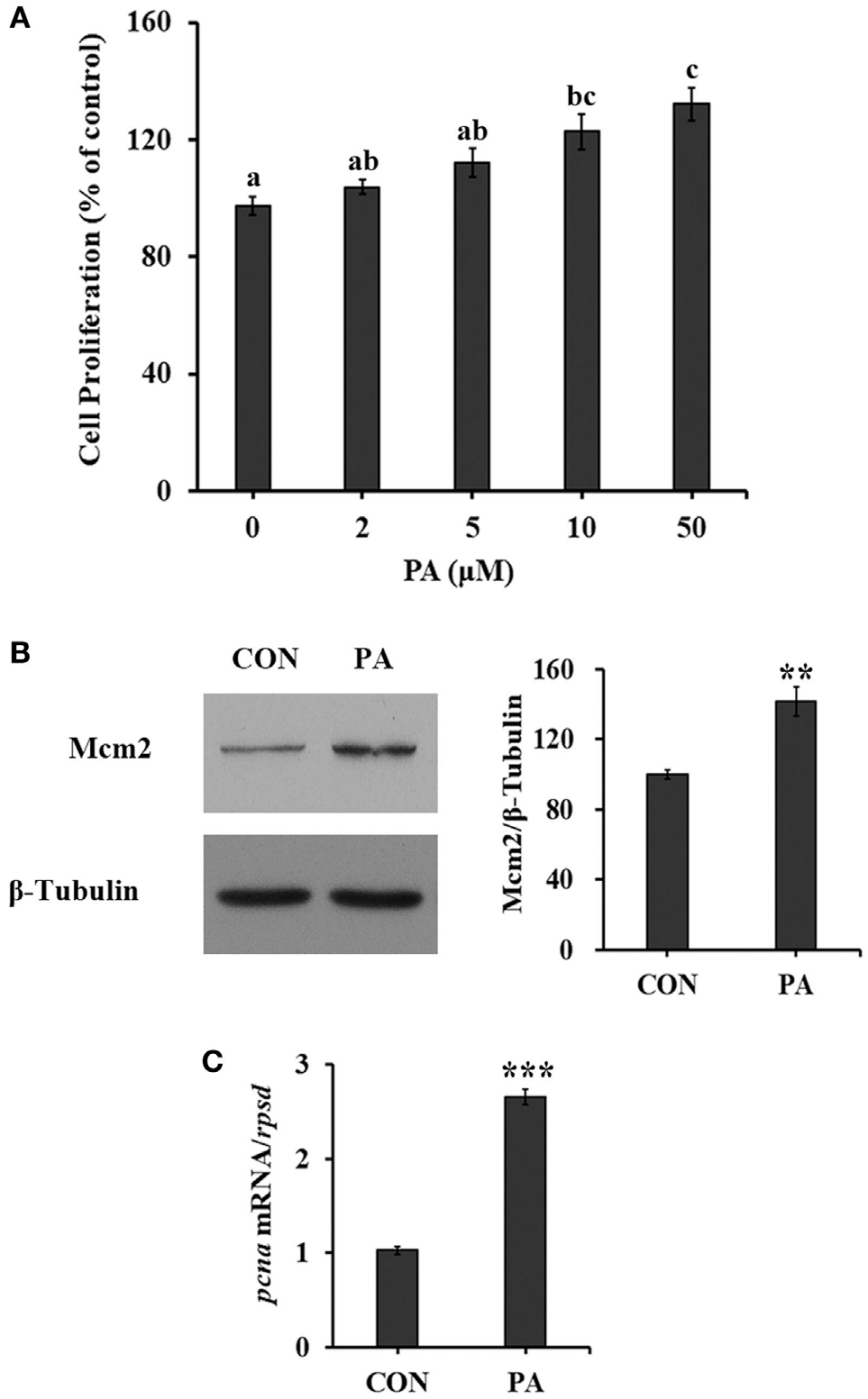
Phosphatidic acid (PA) induced cell proliferation. Serum starved cells were treated with indicated concentrations of PA for 16 h. **(A)** Cell proliferation was evaluated by the CyQUANT Assay Kit after PA treatment (*n* = 6). **(B)** The level of minichromosome maintenance protein 2 (Mcm2) was examined by Western blot after PA treatment and quantitated (*n* = 3). **(C)** The mRNA expression level of *proliferating cell nuclear antigen (pcna)* was quantitated by quantitative real-time PCR after PA treatment (*n* = 6). Results were represented as means with SEs and significance was evaluated by one-way ANOVA followed by Tukey’s multiple range tests in **(A)** (^a,b,c^ means with different letters were significantly different, *P* < 0.05) and independent *t* tests in **(B,C)** (*P < 0.05, **P < 0.01, ***P < 0.001).

The cell proliferation of turbot primary muscle cells was stimulated by PA in a dose-dependent manner (Figure 1A). The cell proliferation rate was increased 35.7 ± 8.8% compared with control after cells were treated with 50 µM PA for 16 h. As biomarkers for cell proliferation, the protein level of Mcm2 was increased 40.8 ± 12.0% (Figure 1B; *P* < 0.01), while the mRNA level of proliferating cell nuclear antigen (*pcna*) was upregulated 166.2 ± 12.9% (Figure 1C; *P* < 0.001) after 10-µM PA treatment. During the experimental treatments, no obvious cellular morphological changes were observed.

### PA Promoted DNA Synthesis and G1/S Phase Transition

**Figure 2:**
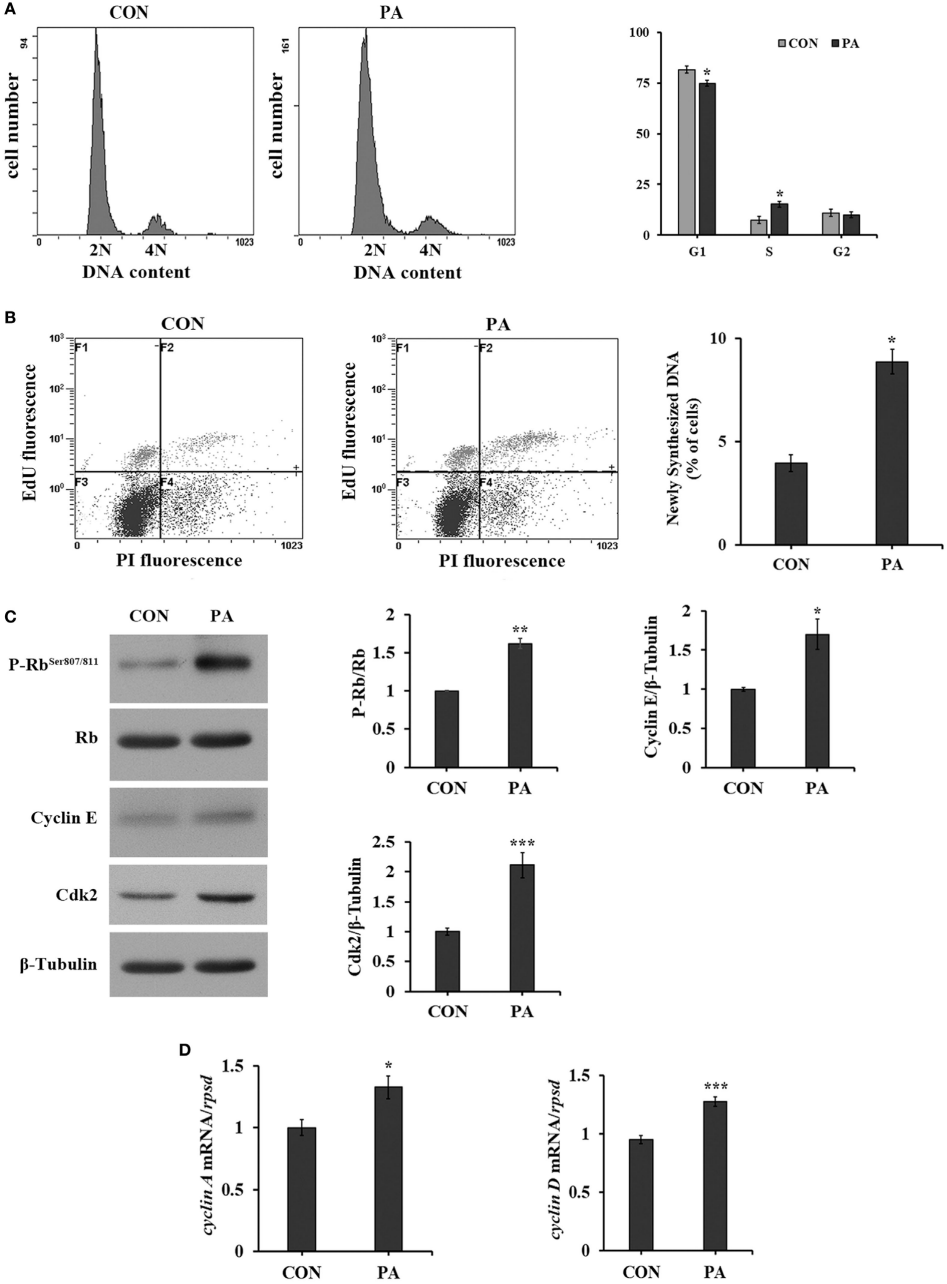
Phosphatidic acid (PA) promoted DNA synthesis and G1/S phase transition. Serum starved cells were treated with 10 µM PA for 16 h. **(A)** The percentages of cells on each phase were analyzed using flow cytometry with PI staining after PA treatment and quantitated (*n* = 3). **(B)** The newly synthesized DNA level was analyzed using flow cytometry with EdU/PI double staining by Click-iT™ Plus EdU Alexa Fluor™ Flow Cytometry Assay Kit after PA treatment (F1, cells in G1 phase; F2, cells in S phase; F3, cells in G0 phase; F4, cells in G2 phase) and quantitated (*n* = 3). **(C)** The levels of total and phosphorylated forms of Rb, cyclin E, and Cdk2 were examined by western blot after PA treatment and quantitated (*n* = 3). **(D)** The mRNA expressions of cyclin A and cyclin D were quantitated by quantitative real-time PCR after PA treatment (*n* = 6). Results were represented as means with SEs and were analyzed using independent *t* tests (*P < 0.05, **P < 0.01, ***P < 0.001).

The cell cycle progression was analyzed by flow cytometry. As shown in Figure 2, the percentage of cells in S phase was increased from 8.2 ± 0.8 to 16.9 ± 1.1% (Figure 2A; *P* < 0.05) after 10-µM PA treatment. The newly synthesized DNA level was significantly increased by 128.2 ± 13.6% by PA treatment (Figure 2B; *P* < 0.05). As a major G1 checkpoint, the Rb protein phosphorylation was increased 62.3 ± 6.6% (Figure 2C; *P* < 0.05). The levels of other G1/S transition facilitators cyclin E and its binding partner Cdk2 were increased 70.0 ± 19.3 and 128.4 ± 29.9%, respectively (Figure 2C; *P* < 0.05). The expressions of their downstream effectors, including *cyclin A* and *cyclin D*, were increased 32.6 ± 15.5 and 34.4 ± 7.7%, respectively (Figure 2D; *P* < 0.05).

### PA Modulated Expression Levels of Muscle Regulatory Factors

**Figure 3:**
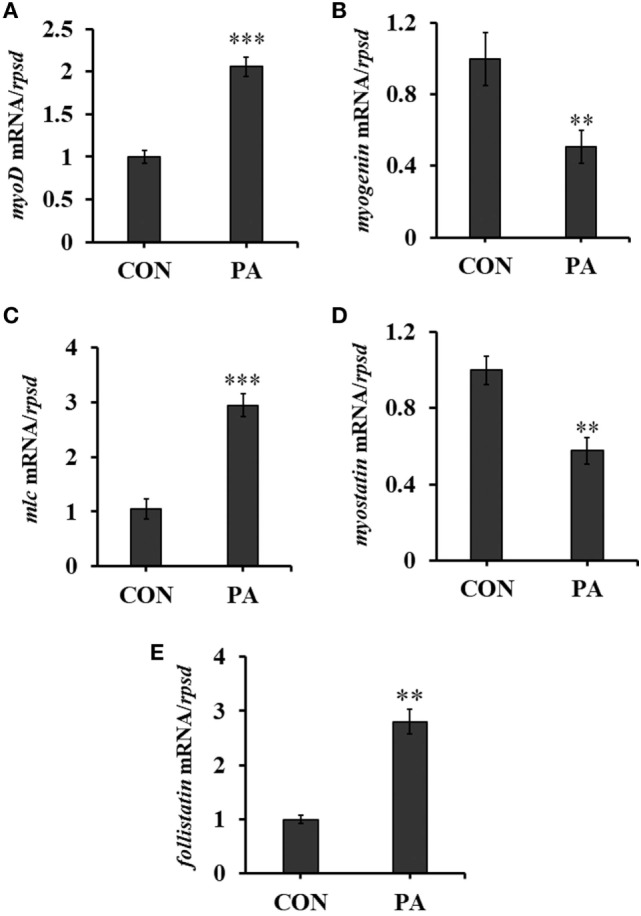
Phosphatidic acid (PA) modulated levels of muscle regulatory factors. The mRNA expressions of **(A)** *myod*, **(B)** *myogenin*, **(C)** *myosin light chain*, **(D)** *myostatin*, and **(E)** *follistatin* were analyzed by quantitative real-time PCR after PA treatment (*n* = 6). Results were represented as means with SEs and were analyzed using independent *t* tests (*P < 0.05, **P < 0.01, ***P < 0.001).

The expression levels of myogenic regulatory factors (MRFs) were quantitated by qRT-PCR. The mRNA level of *myoD* was upregulated 134.7 ± 23.0% while that of *myogenin* was downregulated 41.9 ± 11.1% (Figures 3A,B; *P* < 0.01). The expression of *myosin light chain (mlc)* was also upregulated 105.6 ± 18.2% (Figure 3C; *P* < 0.05). Furthermore, the expression of *myostatin*, a negative regulator of muscle growth was downregulated 45.5 ± 14.4% while that of *follistatin*, the antagonist of *myostatin*, was upregulated 200.5 ± 24.0% significantly (Figures 3D,E; *P* < 0.01).

### PA Induced Cellular Protein Synthesis and G1/S Phase Transition in a TOR-Dependent Manner

**Figure 4:**
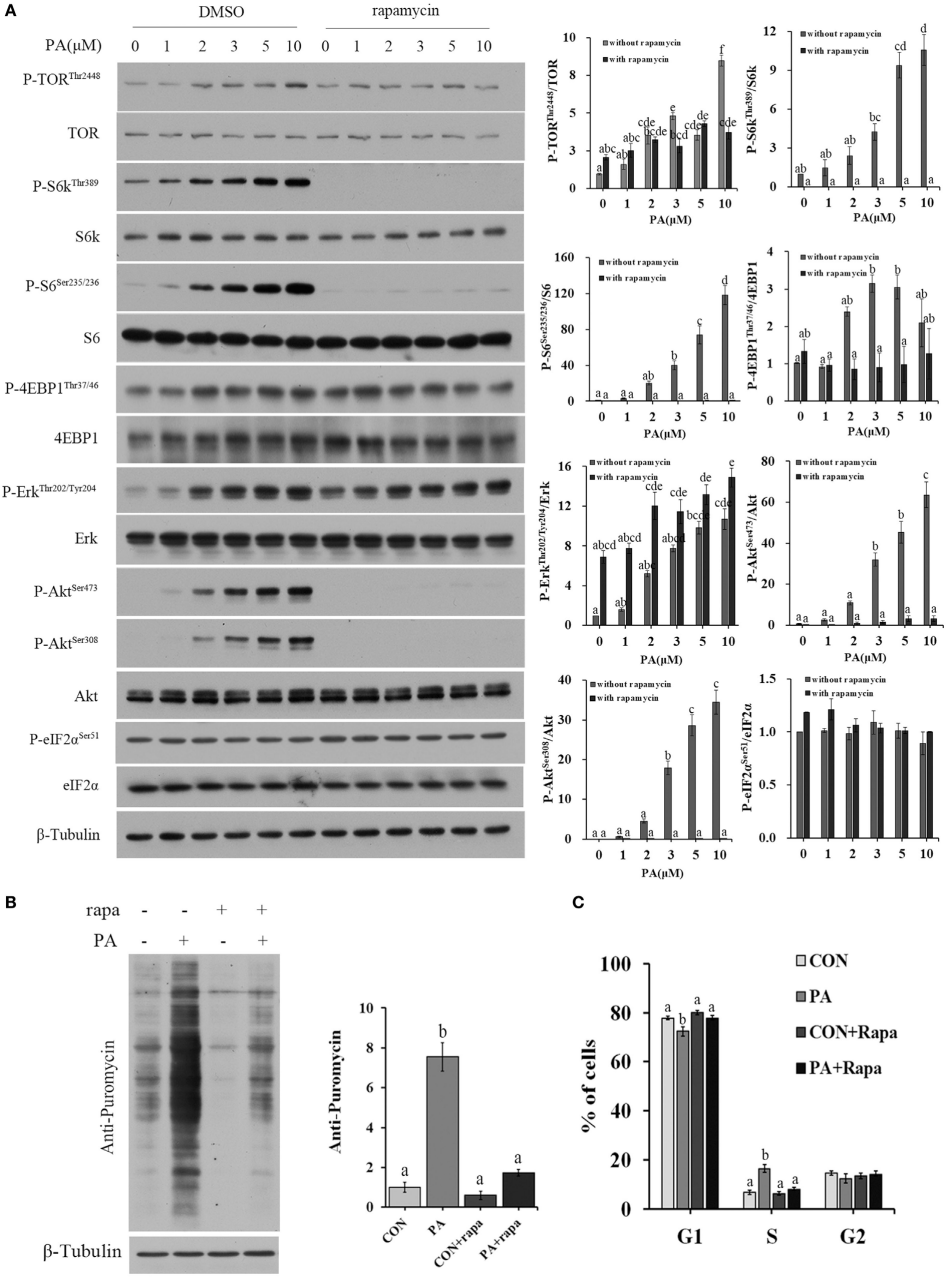
Phosphatidic acid (PA) induced cellular protein synthesis and G1/S phase transition in a target of rapamycin (TOR)-dependent manner. Serum starved cells were treated with indicated concentrations of PA with or without rapamycin for 16 h. **(A)** The levels of total and phosphorylated forms of TOR, S6k, S6, 4EBP1, Erk, Akt, and eIF2α were examined by western blot after treatment and quantitated (*n* = 3). **(B)** Cellular protein synthesis was analyzed by western blot using SUnSET technique after treatment (*n* = 3) and quantitated. **(C)** Cell cycle distribution was analyzed using flow cytometry with PI staining after treatment and quantitated (*n* = 3). Results were represented as means with SEs and significance was evaluated by one-way ANOVA followed by Tukey’s multiple range tests. ^a,b,c,d,e,f^ means with different letters were significantly different, *P* < 0.05.

The activities of major nutrient sensing signaling pathways were also examined. PA treatment increased the phosphorylation levels of Akt (Ser473, Ser308), TOR (Thr2448), 4EBP1 (Thr36/47), S6k (Thr389), S6 (Ser235/236), and Erk (Thr202/204) in a dose-dependent manner (Figure 4A). Rapamycin treatment inhibited all of the above phosphorylations except that of Erk (Figure 4A). The newly synthesized protein rate was increased by 755.0 ± 70.8% in PA group compared with control (Figure 4B; *P* < 0.001). The stimulus effect of PA was diminished to an insignificant level after 10 µM rapamycin treatment. The stimulation of S phase percentage by PA was also diminished by rapamycin (Figure 4C).

### PA Modulated Levels of Intracellular Free Amino Acids, Amino Acid Transporters, Amino Acid, and Protein Catabolism

**Figure 5:**
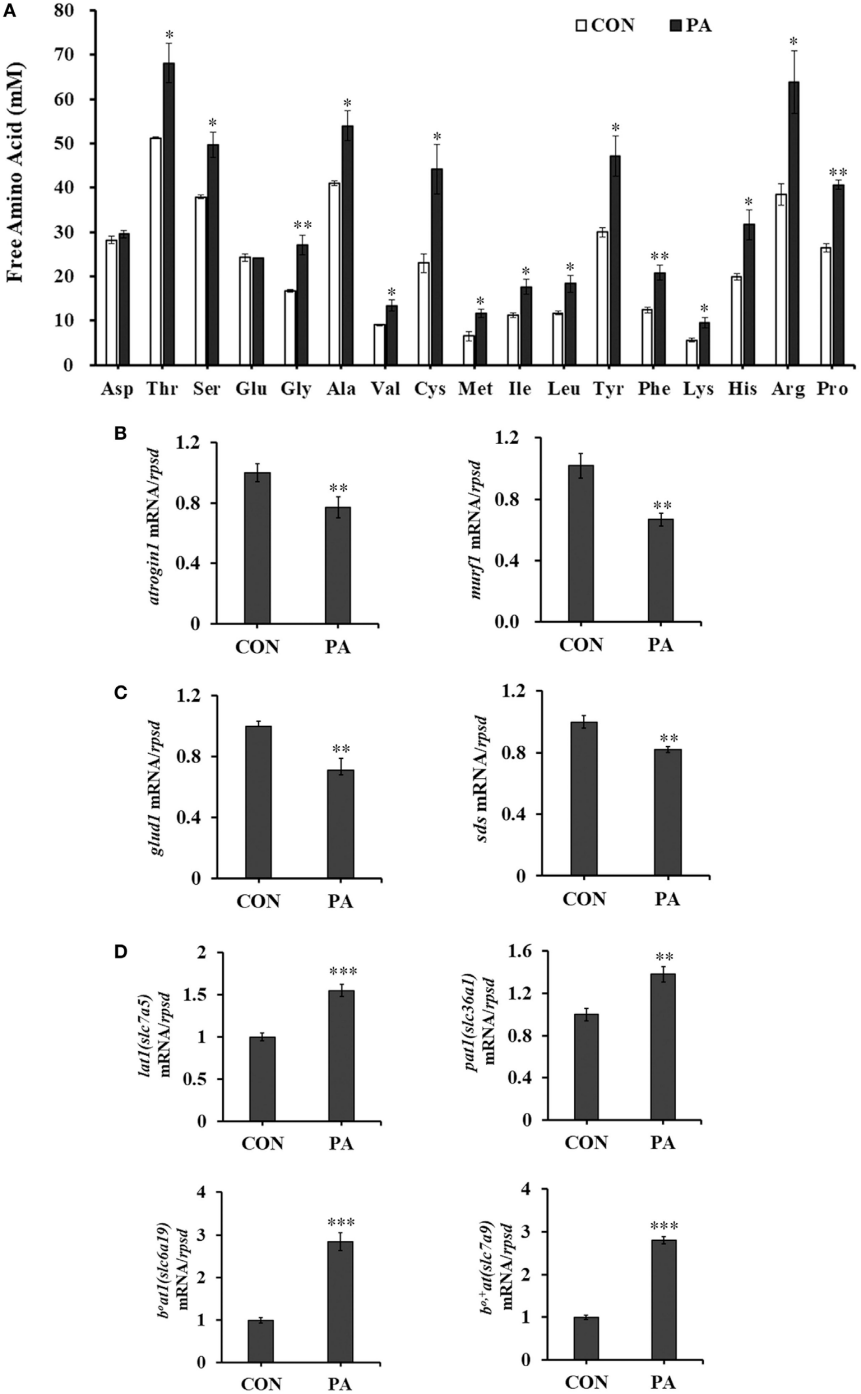
Phosphatidic acid (PA) modulated levels of intracellular free amino acids, amino acid transporters, amino acid, and protein catabolism. Serum starved cells were treated with 10 µM PA for 16 h. **(A)** Intracellular free amino acids concentrations changed after PA treatment (*n* = 3). **(B–D)** The mRNA expressions of *atrogin1, murf1*; *glud1, sds*; *lat1, pat1, b^0^at1*, and *b^0,+^at* were analyzed by quantitative real-time PCR after PA treatment (*n* = 6). Results were represented as means with SEs and were analyzed using independent *t* tests (*P < 0.05, **P < 0.01, ***P < 0.001).

The concentrations of intracellular free amino acids were increased between 31.1 and 92.2% after PA treatment (Figure 5A; *P* < 0.05). Meanwhile, the mRNA expression of protein degradation markers, including muscle atrophy markers *atrogin-1* and *muscle RING finger 1 (murf-1)* were downregulated (Figure 5B; *P* < 0.01) 33.0 ± 7.3 and 40.4 ± 7.0%, respectively. The mRNA expressions of key enzymes in amino acid catabolism, including *glutamate dehydrogenase (glud1)* (67.1 ± 24.3%) and *l-serine ammonia-lyase (sds)* (17.6 ± 4.9%) were also downregulated (Figure 5C; *P* < 0.01). The expression levels of major amino acid transporters, including *lat1* (*slc7a5*, 72.0 ± 17.7%), *pat1* (*slc36a1*, 39.1 ± 14.5%), *b^0^at1* (*slc6a19*, 183 ± 24.1%), and *b^0,+^at* (*slc7a9*, 180.6 ± 13.0%) were increased significantly after PA treatment (Figure 5D; *P* < 0.05).

### PA Influenced Fatty Acid Metabolism, Glycolysis, and Energy Metabolism

**Figure 6:**
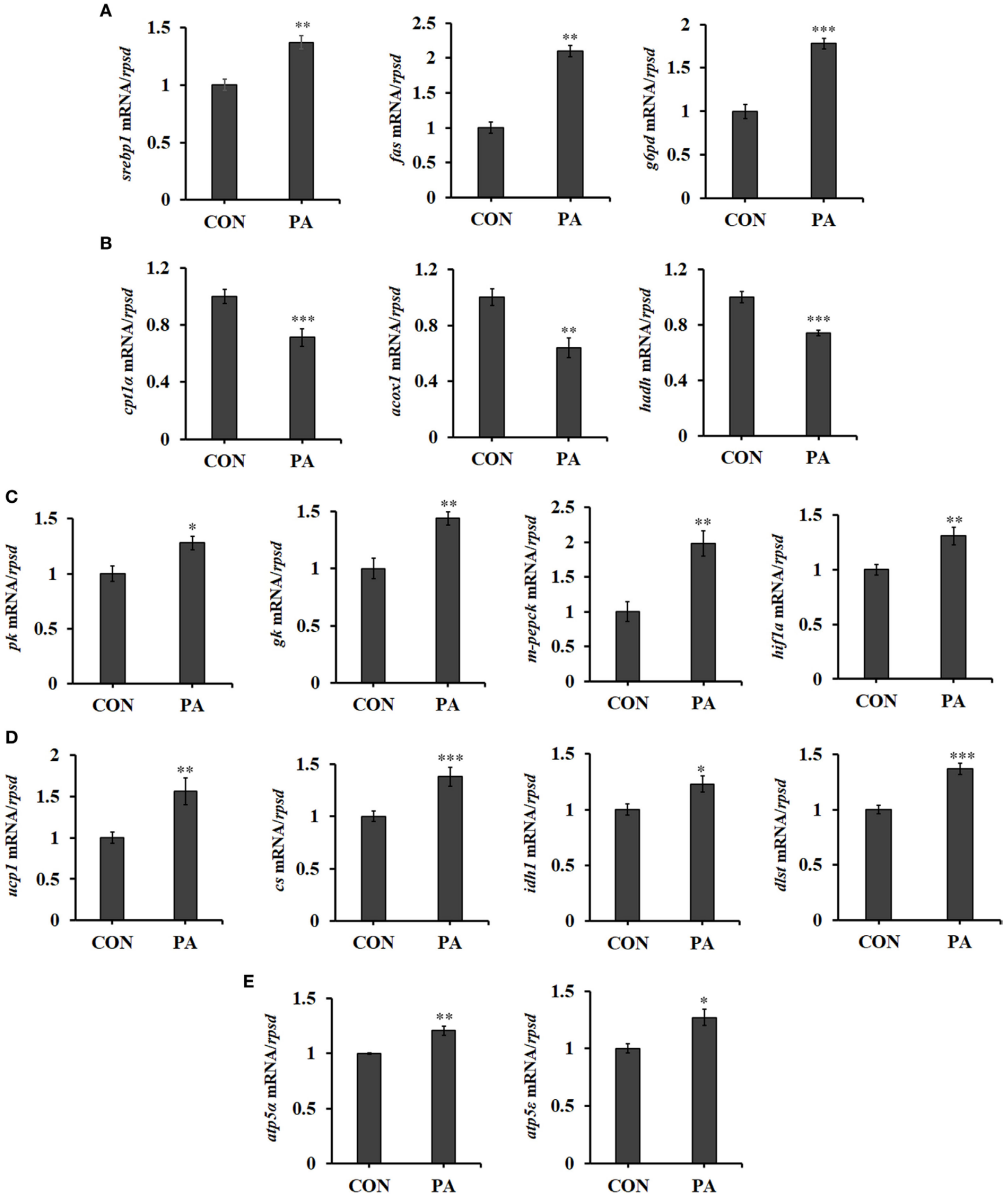
Phosphatidic acid (PA) influenced fatty acid metabolism, glycolysis, and energy metabolism. The mRNA expressions of **(A)** *sterol regulatory element-binding protein 1 (srebp1), fatty acid synthetase (fas), glucose-6-phosphate dehydrogenase (g6pd)*, **(B)** *cpt1*α, *acox1, hadh*, **(C)** *pk, gk, m-pepck, hypoxia-inducible factor 1-alpha (hif1*α*)*, **(D)** *ucp1, cs, idh1, dlst*, **(E)** *atp5*α, and *atp5*ε were analyzed by quantitative real-time PCR after PA treatment (*n* = 6). Results were represented as means with SEs and were analyzed using independent *t* tests (*P < 0.05, **P < 0.01, ***P < 0.001).

The mRNA expression of genes involved in fatty acid synthesis, including *sterol regulatory element-binding protein 1 (srebp1), fatty acid synthetase (fas)*, and *glucose-6-phosphate dehydrogenase (g6pd)* (55) were upregulated after PA stimulation (Figure 6A; *P* < 0.01). Meanwhile, the expression of genes involved in fatty acid β-oxidation (56), including *carnitine palmitoyltransferase 1 isoforms* α *(cpt1*α) (57), *acyl-CoA dehydrogenase (acox1)*, and *3-hydroxyacyl-CoA dehydrogenase (hadh)* (58) were downregulated in PA group (Figure 6B; *P* < 0.01). Furthermore, PA increased mRNA levels of key enzymes in glycolysis, including *pyruvate kinase (pk), glucokinase (gk)*, and *mitochondrial phosphoenolpyruvate carboxykinase (m-pepck)* (Figure 6C; *P* < 0.05). A positive transcription regulator of glycolysis, *hypoxia-inducible factor 1-alpha (hif1α)* (59), was also upregulated (Figure 6C; *P* < 0.01). The expressions of key enzymes involved in energy production, including *uncoupling protein 1 (ucp1), citrate synthesis (cs), isocitrate dehydrogenase1 (idh1)* (60), and *dihydrolipoamide succinyltransferase (dlst)* were upregulated (Figure 6D; *P* < 0.05). The levels of key molecules involved in oxidative phosphorylation were also upregulated in PA group. These included *F-type H* + -*transporting ATPase subunit alpha (atp5*α) and *F-type H* + *-transporting ATPase subunit epsilon (atp5ε)* (Figure 6E; *P* < 0.05).

## Discussion

In this study, PA was found to stimulate cell proliferation in a dose-dependent manner (Figure 1A). This was further confirmed by the increased levels of two cell proliferation markers (Mcm2 and *pcna*, Figures 1B,C) (61, 62) and newly synthesized DNA (Figure 2B). As measured, the percentage of cells entering S phase was increased by PA (Figure 2A). Previous study also demonstrated that inhibition of *de novo* synthesis of PA resulted in G1 cell cycle arrest (46). Mechanistically, the G1/S phase transition requires cyclin D-CDK4/6 complex accumulation (4). The phosphorylation of retinoblastoma protein (Rb) by Cyclin D–CDK4/6 complex can dissociate it from E2F and trans-activates the target genes including cyclins (cyclin A and E) that facilitate G1/S phase transition and S-phase (4, 20, 63). Our data showed that PA induced the Rb phosphorylation and upregulated the expressions of cyclin A, D, E, as well as Cdk2 (Figures 2C,D). This is consistent with what was observed in mammalian cells such as Swiss 3T3 cells (64, 65) and osteoblastic cells (66).

Hyperplasia and hypertrophy in fish muscle growth is tightly regulated by the sequential expression of transcription factors known as MRFs (67, 68). Quiescent satellite cells display no detectable levels of MRFs (69). Upregulation of MyoD precedes the expression of PCNA, a marker for cell proliferation (61). Muscle regeneration is severely impaired in MyoD (−/−) mice and is characterized by an almost complete absence of proliferative myogenic precursor cells (70). On the other hand, the expression of myogenin is associated with myoblast fusion and differentiation (71, 72). Thus, myoblast proliferation and hyperplasia can be inferred by the high expression of *myoD*, whereas *myogenin* expression can be related to myoblast hypertrophy in fish (73, 74). In this study, PA increased the expression level of *myoD* while decreased that of *myogenin* (Figures 3A,B), clearly suggesting its cell proliferative and hyperplastic effects. In gilthead sea bream myocytes, amino acids but not IGF1 stimulated TOR phosphorylation and *myoD* expression. However, IGF1 was able to increase the protein expression of myogenin (28, 29). Lysine limitation decreased expressions of *IGF1, myoD*, and *myogenin* in similar primary muscle cell cultures (75). These results clearly highlighted the distinct roles of biomolecules and signals in fish myogenesis (76). In fish, the accretion of myofibrillar proteins that makes the single most important contribution to fish growth. Myostatin is a TGF-β family member that acts as a negative regulator of skeletal muscle mass, while follistatin counteract its effect (77, 78). Our results showed that the expressions of *myostatin* and *follistatin* were down and upregulated respectively by PA (Figures 3D,E). The increased expression of *mlc* by PA further supported the positive role of PA in muscle hyperplasia and muscle mass production in turbot (Figure 3C).

Muscle growth represents the net balance between protein anabolism and catabolism (79). As the key pathway that governs protein synthesis and anabolism, TOR signaling plays a significant role in muscle growth in mammals (17, 80–82). Inhibition of TOR activity with rapamycin blocks hypertrophy in C2C12 induced myotubes and rat muscles (81). Several studies also demonstrated that TOR activities were closely associated with metabolism and nutrient utilization in fish (31, 55, 83–85). Our previous studies demonstrated that chronic inhibition of TOR by rapamycin significantly inhibited the growth in turbot (34). To date, there has been no report on the effect of TOR potentiation in fish. PA was shown to directly bind to TOR (41) and enhances its signaling both *in vitro* and *in vivo* (39, 45, 46, 86). Here, we demonstrated for the first time that PA addition induced a rapid and dose-dependent activation of TOR signaling in fish cells (Figure 4A). Similar effector was also observed in serum-starved HEK293 cells treated with high dose of PA (41, 45). PA treatment did not influenced the cellular phosphorylation level of eIF2α (Figure 4A), suggesting its effect was not likely cell stress related (87). It was known that starvation suppressed cellular TOR activities and protein synthesis (88). However, supplementation of PA strongly stimulated both of these parameters (Figures 4A,B). The stimulatory effects of PA on both protein synthesis and G1/S phase transition were abolished by rapamycin (Figures 4B,C). These results suggested that the stimulatory effects of cell proliferation and protein synthesis by PA were mediated by TOR signaling. Similar to what was reported in mammalian cells (39, 89), PA was also found to stimulate Erk phosphorylation (Figure 4A). This effect might be acted upstream of TOR (86).

The kinetics of intracellular free amino acids reflects the global output of cellular activities and also provides the critical input to the cellular signaling machinery (90). In this study, the intracellular free amino acids levels were increased after PA treatment (Figure 5A). The balance between catabolic and anabolic processes exerts a homeostatic control on the free amino acid pool (91). PA significantly downregulated the levels of key molecules involved in protein and amino acid degradations, while promoted protein synthesis as discussed above. Furthermore, PA stimulated the expressions of major amino acid transporters, which might facilitate across-membrane transportation of extracellular amino acids for intracellular biosynthesis (92). A rapid increase of amino acid transporters was observed in skeletal muscle following essential AA ingestion in humans and was associated with the muscle protein anabolic response (93). LAT1 (SLC7A5) in particular was reported to mediate large neutral amino acids entry and the downstream activation of intracellular AA sensors such as TOR signaling (94). PAT1 (SLC36A1) was found to mediate the transportation of amino acids such as proline (95) and act as an amino acid transceptor for the activation of TOR signaling in several cell lines (96). In addition, the major neutral amino acid transporter *b^0^at1* (*slc6a19*) (97) and cationic amino acid transporter *b^0,+^at* (*slc7a9*) (98) were identified in turbot muscle in our previous study (99) and were found to be upregulated in the present study (Figure 5D). These data suggested that the intracellular amino acid availabilities, activation of TOR signaling, and downstream anabolic processes were tightly coordinated and regulated.

The effects of PA on the cellular metabolism were also examined. Increased lipogenesis is a common feature shared by most proliferating cells (100). In this study, the expression levels of major lipogenic factors (*fas, srebp1*, and *g6pd*) were upregulated while those of major fatty acid β-oxidation enzymes were downregulated by PA (Figures 6A,B). Glycolysis in proliferating cells provides intermediates for biosynthesis and ATP production (11). The mRNA expressions of major enzymes (*pk, gk*, and *m-pepck*) in glycolysis were all upregulated (Figure 6C) by PA. Thus, increased glycolysis was synchronized with enhancement of anabolism after PA stimulation. Such coordination was mediated though the regulation of TOR activities and its downstream effectors such as HIF1α, which acted upstream of glycolysis (59). ATP is highly demanded for biosynthesis. In fact, mitochondrial activity is enhanced in stimulated lymphocytes when compared with resting lymphocytes, and maximal activity correlates with peak DNA synthesis during S phase (101). Represented by the upregulated expression levels of key enzymes (Figures 6D,E), the tricarboxylic acid cycle and oxidative phosphorylation were stimulated by PA.

In summary, this study demonstrated that supplementation of PA significantly enhanced cell proliferation and anabolism through the activation of TOR signaling in primary muscle cells of turbot. This represents the first report of PA’s effects in fish, given the consideration of disparate growth pattern compared with mammals. Further validation of the effects of PA to promote fish growth will be conducted through growth trials and *in vivo* experiments. Identification of anabolic promoting nutraceuticals like PA should provide new strategies for better nutrition utilization and efficiency in aquaculture.

## Ethics Statement

This study was carried out in accordance with the recommendations of the Animal Care Committee of Ocean University of China. The protocol was approved by the Animal Care Committee of Ocean University of China.

## Author Contributions

GH and KM designed the research. GH, TW, and XW conducted the research, analyzed the data, and wrote the paper. HZ and HJ provided technical assistance and contributed to the preparation of the figures. All authors read and approved the final manuscript.

## Conflict of Interest Statement

The authors declare that the research was conducted in the absence of any commercial or financial relationships that could be construed as a potential conflict of interest. The reviewer JG and handling Editor declared their shared affiliation.
