# Supplementary material for: The Mitotic and Metabolic Effects of Phosphatidic Acid in the Primary Muscle Cells of Turbot (Scophthalmus maximus)
Source: Front Endocrinol (Lausanne). 2018 May 4;9:221. doi: 10.3389/fendo.2018.00221 (PMC5946094; doi:10.3389/fendo.2018.00221)
Supplement: Supplementary file 1 [file Table_1.PDF]

Supplemental Table. The primer pairs sequences used for qRT-PCR.

Gene	Forward Primer(5'-3')	Reverse Primer(5'-3')	Product Size(bp)
pcna	CTCGTACCGCTGCGACAG	CAGAGGGCATCTTCACCAC	251
cyclin A	TGAAGAAATCTACCCCCCTGA	CTCTCCACCTGCTTGCTAACA	189
cyclin D	CTCTGTATCTACACAGACAACTC	GACTCTATTTGCTCCTGACA	389
atrogin1	AGGAGAACTTGCTGCTGTCG	AGATCCAAGCGGTTGAAGG	184
murf1	CTGCCGCTTCGAGGTGAT	TGATGCGTTCGTCTTCGTG	185
glud1	TTCGTCATCCAGGGGTTTG	GCGTTCCAGATACTTCCGT	110
sds	ATGGCAGCGGCTTATTCT	GCTTCACCCTTAGGTCTCG	271
pat1	TCAGTGACAACATCAAGCAGGTG	GAAGGCGGGCAGGAAGAAGAG	140
lat1	GCATCCTCCTGTTGACCTTT	CGGAAGGTTCTTGTACGGGTC	293
$b^{0,+}at$	GGGCTTTGGGCTTATGATGGATG	TGGAGACAACAGCAGTTCAGTGG	180
$b^{0}at1$	AGACTCTCAACACCTCCGAAGC	AGCCTTTCCTGTGGTCTCAATCC	131
myoD	GCTGCACCTCCACCACCA	GTTGACCTTGCCGAGCC	181
myogenin	CGGGTTGGGTCGGTAGTGTA	CGGCGTCTGAAGAAGGTGAA	198
mlc	ATGGTGAGGAAGACGGTGAA	AGGCAGGCAGCAGGAGACAG	235
myostatin	GAAGACTTTGGCTGGGACT	AGAGCATGTTGATGGGTGA	183
follistatin	GTGCTCCAGACTGCTCCAA	AGATGATCCCGTCGTTTCC	288
srebp1	GCCATTGACTACATCCGTTAC	CATCAGCCTGTCCATCTACTTC	136
fas	GGCAACAACACGGATGGATAC	CTCGCTTTGATTGACAGAACAC	195
g6pd	ACGTGCCAGGAGACATATTTAGT	AACTCATCACTGCGGACAAAG	250
cpt1α	ATGGGAAGAGTGGACTGAATG	GCTGGAAGGCATCTGTGG	96
acox1	AGTCCTCGCCCAGCTTTACT	GGCTTCACATAGGTTCCGTCT	240
hadh	TTCACAATGAATCCAGGCG	TCATCGGAACCTCGGCAACAAG	112
hif1α	AGGGACAGGTCAGCACAGG	CATCATCGGTCTGCTCCAAAG	189
pk	TGGATACGCTGAAGGAGATG	ACGCACGTTCTTGATGGTC	236
gk	CGACACGAGGACATTGACAAG	CCAACAATCATCCCGACTTCAC	218
m-pepck	CTGGTCTACGAGTCGTTCAAC	GGTAGTCGCCAAAGTTGTAGC	154

ucp1	CAAAGTGGCAAGGCAGAT	GCGTGGACTATGGAAAGG	140
cs	GTGACCAACAGCCAGAAG	GAACAGACCAGGATCAAAGAAC	246
idh1	TCCTGGAAGATGTCTTTGAAGCG	GAGAGCGGAGAACCCGTTAAATATG	202
dlst	CCGTTGCTGATGGTGAAG	CCCGCTGTCAATGCTGTA	236
atp5α	TTGGTCCCCATTGGTCGT	GCGTTTCTGGTTGATGATTGTGTC	100
atp5ε	GGAAGCCAACGTCAAAGTGG	ACAGGGGCAGGACCTCAACT	102
rpsd	CTGCTGTTCCCTAAAGAGTTCG	GAGCCGTGTAGTTCAGGGTCT	151

Abbreviations and GenBank Accession No.:

pcna, *proliferating cell nuclear antigen*, EU711051.1; *myostatin*, EF683115.1; *fas*, *fatty acid synthetase*, KC189927; *cpt1a*, *carnitine palmitoyltransferase 1 isoforms A*, KC189926; *acox1*, *acyl-CoA oxidase 1*, KC189925; *pk*, *pyruvate kinase*, DQ848903; *gk*, *glucokinase*, JX678944; *m-pepck*, *mitochondrial phosphoenolpyruvate carboxykinase*, KC149516.1; *rpsd*, *RNA polymerase II subunit D*, DQ848899.1.

Partial sequences of some target genes in turbot were obtained through a degenerate PCR strategy in this study, including *cyclin A*, MH174968; *cyclin D*, MH174965; *atrogin1*, MH174969; *murf1*, *muscle RING-finger 1*, MH174966; *glud1*, *glutamate dehydrogenase 1*, MH174955; *sds*, *L-serine or L-threonine ammonia-lyase*, MH174956; *pat1*, *proton-coupled amino acid transporter 1*, MH174961; *lat1*, *L-type amino acid transporter 1*, MH174962; $b^{0,+}at$, $b^{0,+}$*-type amino acid transporter*, MH174963; $b^{0}at1$, b^{0}*-type amino acid transporter 1*, MH174967; *myoD*, *myogenic differentiation antigen D*, MH174948; *myogenin*, MH174947; *mlc*, *myosin light chain*, MH174950; *follistatin*, MH174949; *srebp1*, *sterol regulatory element-binding protein 1*, MH174964; *g6pd*, *glucose-6-phosphate dehydrogenase*, MH174959; *hadh*, *3-hydroxyacyl-CoA dehydrogenase*, MH174957; *hif1α*, *hypoxia-inducible factor 1-alpha*, MH174960; *ucp1*, *uncoupling protein 1*, MH174946; *cs*, *citrate synthesis*, MH174951; *idh1*, *isocitrate dehydrogenase1*, MH174952; *dlst*, *dihydrolipoamide succinyltransferase*, MH174953; *atp5α*, *F-type* H^{+}*-transporting ATPase subunit alpha*, MH174954; *atp5ε*, *F-type* H^{+}*-transporting ATPase subunit epsilon*, MH174958. These accessions were pending for NCBI approval during the manuscript preparation.
